# Mitochondrial concept of leukemogenesis: key role of oxygen-peroxide effects

**DOI:** 10.1186/1742-4682-5-23

**Published:** 2008-11-11

**Authors:** Boris N Lyu, Sanzhar B Ismailov, Bolat Ismailov, Marina B Lyu

**Affiliations:** 1 Scientific Center for Anti-Infectious Drugs, Almaty, Kazakhstan

## Abstract

**Background and hypothesis:**

The high sensitivity of hematopoietic cells, especially stem cells, to radiation and to pro-oxidative and other leukemogenic agents is related to certain of their morphological and metabolic features. It is attributable to the low (minimal) number of active mitochondria and the consequently slow utilization of O_2 _entering the cell. This results in an increased intracellular partial pressure of O_2 _(pO_2_) and increased levels of reactive oxygen (ROS) and nitrogen (RNS) species, and a Δ(PO – AO) imbalance between the pro-oxidative (PO) and antioxidative (AO) constituents.

**Proposed mechanism:**

Because excessive O_2 _is toxic, we suggest that hematopoietic cells exist in a kind of unstable dynamic balance. This suggestion is based on the idea that mitochondria not only consume O_2 _in the process of ATP production but also constitute the main anti-oxygenic stage in the cell's protective antioxidative system. Variations in the mitochondrial base capacity (quantity and quality of mitochondria) constitute an important and highly efficient channel for regulating the oxidative stress level within a cell.

The primary target for leukemogenic agents is the few mitochondria within the hematopoietic stem cell. Disturbance and weakening of their respiratory function further enhances the initial pro-oxidative state of the cell. This readily results in peroxygenation stress, creating the necessary condition for inducing leukemogenesis. We propose that this is the main cause of all related genetic and other disorders in the cell. ROS, RNS and peroxides act as signal molecules affecting redox-sensitive transcription factors, enzymes, oncogenes and other effectors. Thereby, they influence the expression and suppression of many genes, as well as the course and direction of proliferation, differentiation, leukemogenesis and apoptosis.

Differentiation of leukemic cells is blocked at the precursor stage. While the transformation of non-hematopoietic cells into tumor cells starts during proliferation, hematopoietic cells become leukemic at one of the interim stages in differentiation, and differentiation does not continue beyond that point. Proliferation is switched to differentiation and back according to a trigger principle, again involving ROS and RNS. When the leukemogenic Δ_L_(PO – AO) imbalance decreases in an under-differentiated leukemia cell to the differentiation level Δ_D_(PO – AO), the cell may continue to differentiate to the terminal stage.

**Conclusion:**

The argument described in this article is used to explain the causes of congenital and children's leukemia, and the induction of leukemia by certain agents (vitamin K3, benzene, etc.). Specific research is required to validate the proposals made in this article. This will require accurate and accessible methods for measuring and assessing oxidative stress in different types of cells in general, and in hematopoietic cells in particular, in their different functional states.

## Background

According to the general oxygen-peroxide concept of carcinogenesis, the primary targets for damage by carcinogenic agents and factors are mitochondria [1]. They consume up to 95–99% of the free oxygen (O_2_) that enters the cell, which is known to be toxic in excess. In this sense, mitochondria perform an anti-oxygen function and constitute the main stage in the protective hierarchical antioxidative system of the cell. This system obviously arose during the course of evolution as organisms adapted to the gradually increasing O_2 _content of the earth's atmosphere. This antioxidative function of mitochondria is usually omitted from accounts or simply ignored by investigators, who emphasize the main role of these organelles as ATP generators.

Any failure of mitochondrial activity must decrease the amount of O_2 _they utilize and consequently increase the partial pressure of O_2 _(pO_2_), i.e. cause intracellular hyperoxia. This induces a proportional increase in the formation of reactive oxygen (ROS) and nitrogen (RNS) species, and in the peroxygenation of different intracellular constituents and structures. These changes are aggravated if both the quantity and quality (i.e. activity) of mitochondria decline. Such disturbances are precisely those observed in neoplastic cells. The argument of the present paper hinges on this major question: why and how does peroxygenation stress, the negative consequences of which have long been well-known, occur in cells? Those consequences have been described in many publications, not only those devoted to carcinogenesis [1].

Thus, both the presence of mitochondria and changes in their quantitative-qualitative parameters constitute an important mode of regulation of the oxygen-peroxide state (peroxygenation stress) and the signaling pathways that depend on it. Considering that mitochondria as dynamic structures are subject to fusion and fission, which alter the mitochondrial base "capacity", this channel of regulation becomes especially efficient [2].

In developing this argument, we first propose the following hypothesis. During the course of evolution, a sequence of "specialized" ranges of intracellular Δ(PO – AO) imbalances has been established between pro-oxidative (PO) and antioxidative (AO) constituents as a way of adapting to increasing oxygen-peroxide levels. Each range became associated with a particular complex biochemical process, perhaps necessarily so. A specific consequence is that several ranges of Δ(PO – AO) imbalance can be generated during the postnatal ontogenesis period. These ranges are distinguished by the particular PO and AO values established under defined conditions; the precise cell state attained corresponds to and/or depends on these values. Within the limits of the Δ_P_(PO – AO) and Δ_C_(PO – AO) imbalance ranges, proliferation (oxidative mitogenesis of a normal non-tumor cell) and carcinogenesis predominate, respectively. At the Δ_Cy_(PO – AO) imbalance, cytolysis occurs; and within the Δ_A1_(PO – AO) and Δ_A2_(PO – AO) imbalance ranges, apoptosis of the A1 and A2 types ensue, respectively. These phenomena mirror evolutionary origins and are seemingly paradoxical: apoptosis of tumor cells is induced by both antioxidants (A1 type) and prooxidants (A2 type) [1,3]. By "range of imbalance" we mean all possible values (not a single value) within the limits of the range. In their abbreviated forms, these "specialized" imbalances are presented as the following sequence:

(a) Δ_P _< Δ_A1 _< Δ_C _< Δ_A2 _< Δ_Cy_

Aging of cells most probably occurs within the Δ_Ag_(PO – AO) imbalance range, which lies between the Δ_P _and Δ_A1 _imbalances. When this range is added, sequence (a) appears as follows:

(b) Δ_P _< Δ_Ag _< Δ_A1 _< Δ_C _< Δ_A2 _< Δ_Cy_

By evaluating and considering the inequalities summarized in (b), we have formulated general propositions about the oxygen-peroxide concept of development, aging, age-related pathologies, carcinogenesis and programmed cell death [1,3]. Mitochondria occupy a central place in the sequence of inequalities since they are critical for the life and death of a cell.

Given the PO and AO constituents of all the imbalances described above, we identify certain *integral *"pro-quantitative" indicators: steady-state (bound) levels of prooxidants and antioxidants, and/or their production rates at the time that they participate in the induction of proliferation, aging, carcinogenesis or apoptosis.

The development of leukemia induced by radiation or other leukemogenic agents is known to be related to the malignant systemic hematopoietic tissue disease, dyshematopoiesis. This pathology is manifest in the proliferation of immature pathological cell elements; it has various types, distinguished by the predominance of different elements. The most widely recognized kind arises when most of the real target cells are pluripotent stem hematopoietic cells (PSHC) and the precursors of different blood cell lines. It is not yet known why these cells are particularly sensitive to different leukemogenic factors; opinions about this matter differ.

A preliminary attempt [4] to use the abovementioned "oxygen-peroxide" concept to understand the mechanism of leukemogenesis seemed reasonable but raised questions. Why and how does such a state occur in PSHC? These questions refer to issues of principle. Of course, it will be desirable to find answers consistent with the "oxygen-peroxide" hypothesis of leukemogenesis. It would be natural to suppose that the mechanism by which neoplastic hematopoietic stem cells are transformed into leukemia cells has features generally similar to, and not fundamentally different from, the malignant transformations of non-hematopoietic cells. The leukemogenesis literature that we have been able to access shows that there really are significant similarities, but at the same time there are important differences. Some of our ideas about this are described below. Before returning to this topic, however, we will elaborate the general reasoning in support of our proposal in the hope of attracting scientific attention to it.

### Oxygen-peroxide effects and the mechanism of leukemogenesis

The "oxygen-peroxide" concept of leukemogenesis is based on the following assumption. PSHC and erythrocyte precursors contain the minimally required number of mitochondria, sufficient only to support a stable Δ_P_(PO – AO) imbalance within them, and undergo practically unlimited proliferation (oxidative mitogenesis). This opinion is confirmed only in single publications. Thus, the authors of [5] demonstrated that the low levels of mitochondrial respiratory chain components in hematopoietic stem cells correlate with the low O_2 _consumption by them. Therefore, such cells must be relatively hyperoxic, and distinctively hypersensitive to radiation and other insults that aggravate the oxygen-peroxide situation.

**Figure 1:**
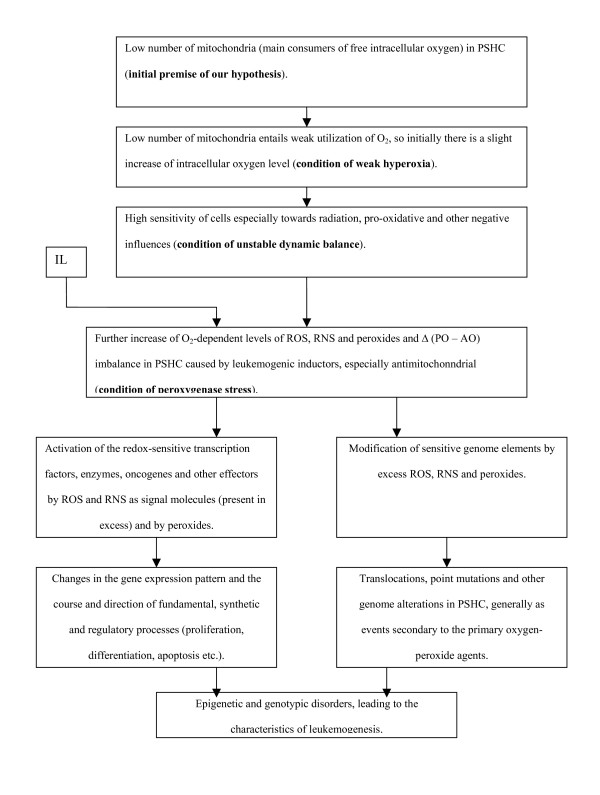
**Structure functional block diagram of mitochondrial (oxygen-peroxide) concept of leukemogenesis.** IL – inducers of leukemogenesis; for other designations see text.

According to our understanding, the required (minimal) number of mitochondria in specialized cell types is naturally adjusted to utilize intracellular O_2 _in order to effect certain basic, non-energetic functions that are also useful for the cell, including synthetic functions. But for this very reason, such cells exist in a state of unstable dynamic balance. Even with an insignificant negative disturbance, mitochondrial respiration readily elevates the Δ(PO – AO) imbalance to pathological levels. In particular, PSHC face an enhanced risk of disease, leaving the normal hematopoiesis pathway, when the normal Δ_P_(PO – AO) imbalance rises to the leukemogenic level Δ_L_(PO – AO); this is similar in essence (in terms of values and evoked consequences) to the "carcinogenetic" imbalance Δ_C_(PO – AO) in non-hematopoietic cells. At Δ_L_(PO – AO) in PSHC both epigenetic and genotypic abnormalities are more probable (see figure 1). For this major reason, the frequency of e.g. spontaneous leucosis increases. Thus, a study of AKR mice revealed an increased frequency and speed of development of leucosis under the influence of small and ultra-small doses of radiation (1.2–2.4 cGy) [6].

The finding that mitochondria are subject to significant negative changes during leucosis transformation in PSHC and bone marrow hemato- and lympho-poiesis precursor cells agrees with our assumption. For instance, according to well-established information [7,8], these changes are mainly qualitative in patients with acute lymphoblast leukemia (ALL) and acute myeloid leukemia (AML), and consist in the following: the number of irregular-shaped mitochondria grows, the mitochondrial matrix and membranes are damaged, cristae are disorganized, etc. By these indicators, leucosis cells do not differ from neoplastic cells of other types. Other authors have reported similar findings. For example, neoplastic cells in AML patients have mitochondria that vary in size and form and show reduced numbers of cristae. These common ultrastructural changes correlate with abnormal metabolic functions such as impaired intra-mitochondrial protein synthesis [9]. ALL patients have more disorganized mitochondria than AML patients; the degree of mitochondrial disorder is greater in patients with bad prognoses [10]. It should be noted that our hypothesis – that the oxygen-peroxide aspect of mitochondria is involved in leukemogenesis – was not considered in the publications cited here, so our proposals on this matter are novel.

A striking feature of leucosis is the bimodal age distribution of its frequency: it primarily affects patients older than 55 years and children under 10 [11]. To date, there has been no satisfactory explanation for the enhanced risk of leucosis in young children. We suggest that in some children the hematopoietic tissue cells and immature hemocytes (blood cells) have excessive Δ(PO – AO) imbalances. This is most likely to be connected to a mitochondrial base insufficiency or mitochondrial deficiency and may have a genetic cause. Individuals who possess such defects must be more predisposed to leukemogenesis, and children in this category become vulnerable to leukemia. Spontaneously, or under the influence of even small doses of prooxidative agents, particularly radiation, leukemia can develop comparatively easily in them.

The same approach may contribute to understanding of the similarly unexplained phenomenon of congenital leukemia. Here, the problem is probably related to the fact that pre-leucosis changes occur in some cases during the prenatal ontogenesis period. These changes develop into the clinical forms of leucosis under the influence of certain postnatal factors [12]. Briefly, our version of this concept is as follows. The low levels of respiratory chain components in PSHC mitochondria correlates, as mentioned above, with weak O_2 _consumption [5]. This feature is apparently also characteristic of the precursors of various blood cell lines. Therefore, increased levels of pO_2 _and peroxygenation stress are imposed on all these cells by the weak mitochondrial capacity. In these different cell lines, which are quite heterogeneous in composition, this leads to an immediate increase of the normal "proliferative" imbalance Δ_P_(PO – AO) to the Δ_L_(PO – AO) level required for leukemogenesis. Within this imbalance range, ROS and peroxide products may modify many intracellular structures, including DNA. It is probably for this reason that chromosome translocations characteristic of children's leucosis are already apparent during normal embryonic development, producing latent leucosis clones [12].

In old age, leucosis apparently develops for the same principal reasons, the only difference being that mitochondrial deficiency and sporadic mutations of mitochondrial DNA in hematopoietic cells are extended in time in the course of ontogenesis and accumulate slowly, acting simultaneously as the aging factor [1].

Other features of the incidence of leucosis in new-born children after administration of vitamin K as an anti-hemorrhagic factor are also of interest. This topic is discussed in [13], which presents the arguments of those who deny that vitamin K is implicated in the induction of leucosis. In our view, a crucial point is that the group K vitamins, specifically vitamin K_3 _(menadion), are able to cause oxidative stress in cells [14]. In PSHC and blood cell line precursors, the prooxidative action of these vitamins should be leukemogenic, in accordance with the oxygen-peroxide concept discussed in this paper. The lack of unambiguous results after vitamin K treatment may be attributable to other conditions of which no account has been taken; this point requires additional research.

Among of the observations supporting the oxygen-peroxide mechanism of leukemogenesis, we should also mention the so-called "benzene" leucosis, which ultimately proves to be ROS-inducible. According to the data in [15], phenolic metabolites of benzene enter the bone marrow and are converted to semiquinone radicals and quinones. ROS formed from these derivatives affect tubulins, histones, topoisomerase II and other proteins linked to DNA, and DNA itself. Since carcinogenic phenolic metabolites of benzene (phenol, hydroquinone, catechol, etc.) are widespread in the environment, they could cause "spontaneous" leucosis in a human by a mechanism such as that described here. However, in this variant of "chemical leucosis", a peroxidase-carcinogenesis status is also created in the bone marrow cells. This results from the inactivation by benzene metabolites of respiratory enzymes and mitochondrial DNA. The latter is known to be quite sensitive to chemical carcinogens [16].

The above-described bioenergetic features of PSHC and blood cell line precursors suggest that such a mechanism for the induction of leukemogenesis, involving hematopoietic cells affected by agents in the microenvironment, is plausible. The proposal concerns reticular cells forming reticular tissue, which in turn constitutes the basis of hematopoietic organs. The reticulo-endothelial cells have high phagocytic capacity. In the presence of agents such as colony-stimulating factors, reticular cells can turn into active macrophages. In this role, they produce ROS and are obviously readily capable of increasing the Δ_P_(PO – AO) imbalance in adjacent (and already comparatively hyperoxic) hematopoietic cells to the leukemogenic level, Δ_L_(PO – AO). In other words, realization of the oxygen-peroxide mechanism of leukemogenesis by macrophages of the hematopoietic organ itself is quite probable. This inference is consistent with our view [17] of the "macrophagous" mechanism of carcinogenesis induced by foreign bodies.

### Other consequences of excessive peroxygenation in leukemia cells

The lipid peroxide (LPO) level in leukemogenesis is significantly enhanced because of direct free-radical oxidation of lipids, which is made likely by a weak mitochondrial capacity and therefore incipient intracellular hyperoxia. In addition, there is an abnormally high density of blood vessels in the bone marrow of patients with acute myeloleukemia or lympholeukemia or with chronic myeloleukemia [18]. Neovascularization is another facet of the oxygen-peroxide mechanism of leukemogenesis. However, judging from the data in the literature, a significant part of the excessive LPO in leukemia cells is also produced by lipid peroxidation processes that are normal parts of the mitogenic cascade. This is clearly apparent when these processes and the effects dependent on them are suppressed.

In this respect, the facts appear to demonstrate that 5-lipoxygenase (5-LOX) inhibitors exert a powerful anti-proliferative action on malignant human hematopoietic cell lines. Thus, while specific lipoxygenase metabolites of arachidonic acid – B_4 _and D_4 _leukotrienes – stimulate proliferation of the malignant lines К-562, EM-2, HL-60 and U-937, a specific 5-LOX inhibitor, Piriprost, causes a reversible 95% inhibition of such proliferation [19]. Activators of 5-LOX, specifically peroxides of fatty acids, increase the tendency of these cells to transform because they reliably enhance the "leukemogenic" Δ_L_(PO – AO) imbalance in them. According to radioimmunoassay data [20], there is increased expression of cyclooxygenase-2 (COX-2) in patients in the chronic stage of chronic myeloblastosis. This increased expression is associated with a low survival rate. These facts are also mostly accounted for by our proposed mechanism: either COX-2 or LOX, participating in arachidonic acid oxidation and ROS formation, contributes to increasing the oxidative stress level in cells. Consequently, COX-2 can serve as an enzymatic basis for the negative "leukemogenic" values of Δ_L_(PO – AO).

Excessive peroxygenation processes are also toxic to intranuclear structures and leukemia cell functions. The targets for ROS and LPO products are DNA and nuclear proteins, particularly non-histone proteins. For example, in children with ALL, changes in basic nitrogens typically induced by hydroxyl radicals are identified in lymphocyte DNA [21]. In this context, it is particularly interesting that there is a statistically greater frequency of p53 anti-oncogene mutations in hematological than non-hematological neoplasms. According to one account, the difference may be related to the absence of hypoxia in most hematological neoplasms [22]. We do not reject that explanation, but suggest that it can be made more specific by invoking the ROS dependence of p53 mutation. A number of publications [23,24] have reported that mutation of the p53 gene in some non-hematological tumor cells is actually caused by oxidative stress. Such stress is likely to be more marked in malignant hematological cells, particularly leukemia cells, in view of their mitochondrial-energetic features. This should make p53 gene mutations more frequent under Δ_L_(PO – AO) than under Δ_C_(PO – AO) conditions.

Moreover, enhanced peroxygenation conditions could also explain the occurrence of multiple significant changes within the nuclear genome during leukemogenesis. The following phenomena are most frequently observed: different translocations of genetic elements, deletions, point mutations, etc. We think it important to stress our alternative "non-genetic" opinion: these genetic changes do not serve *in toto *as the original cause of leukemogenesis; on the contrary, they are mainly *secondary *to the effects of primary DNA-modifying agents. As we have said, the latter include excessive ROS, RNS and some peroxides [1,25] formed under the influence of different agents and factors, specifically leukemogenic ones. Hematopoietic cells are particularly sensitive to such effects from the outset because of their mitochondrial-energetic features (see above). This is precisely why prooxidant agents lead comparatively easily, directly or indirectly, to negative rearrangements of the nuclear genome in such cells.

The oxygen-peroxide concept of leukemogenesis explains many features of the protective actions of various antioxidants. Among the latter, we draw particular attention to resveratrol, a phenol antioxidant that is a natural component of grape skins. Depending on the dose, resveratrol suppresses the growth of THP-1 human monocytic leukemia cells [26]. It also displays anti-leukemia activity with respect to mouse (L1210) and human (U937, HL-60) leucosis cells, suppressing their proliferation. Furthermore, resveratrol inhibits the proliferation of normal hematopoietic precursor cells, but this effect is partially reversible while its action on leukemia cells is irreversible [27]. Resveratrol is not only an anti-leukemia agent but also a universal anti-carcinogenic agent. This indirectly supports the view that these processes are rooted in the general oxygen-peroxide mechanism we have proposed.

2-chloro-2'-deoxyadenosine has been promoted as an efficient new anti-leucosis drug. Its characteristic feature is its early action on mitochondrial functions and mitochondrial DNA content, as shown within ≤ 7 days in cultured CCRF human leukemia cells [28]. Intracellular lactic acid formation was used in that study to trace changes in oxidative phosphorylation and mitochondrial dysfunction. A brief incubation with 2-chloro-2'-deoxyadenosine increased the amount of lactate after a 12-hour exposure, in parallel with cytotoxicity. From our point of view, these results are attributable to an increase in the Δ(PO – AO) imbalance from the leukemogenic level to the A2 apoptosis level, or immediately to cytolysis, primarily caused by reduced mitochondrial respiration and hyperoxia inside the leukemia cells aggravating the shifts already present. Basically the same effects should be caused by any other agent and factor that enhances oxidative stress in leukemia cells. Among such factors are apparently those that induce apoptosis in HL-60 [29] and U-937 [30] cells. In contrast, reduction of the Δ_L_(PO – AO) imbalance in these cells should lead to A1 apoptosis. A possible example is illustrated in [31].

The proposed mitochondrial concept of leukemogenesis is summarized in the block diagram (figure 1).

### Differentiation of leukemia cells: problem analysis and suggestions

Another aspect of the topic under discussion still remains difficult to understand: the connection between the processes causing leukemogenesis and the differentiation of hematopoietic cells during early development. The fact that this differentiation process is often enhanced during leucosis transformation testifies to the existence of such a connection [32]. It would seem that in the course of normal differentiation, just as in case of non-hematopoietic cells, energy requirements should lead towards the development of increased numbers of well-differentiated mitochondria [2,33], and this should effectively increase the intensity of mitochondrial respiration and concomitantly reduce the intracellular Δ_P_(PO – AO) imbalance and POL level. The latter is needed for normal oxidative mitogenesis in immature cellular elements of the hematopoietic system.

However, events may develop in other directions during the differentiation of hematopoietic and leukemia cells. This is implied by the following major difference: most types of neoplasm lose control over their own fission, but in the case of leucosis, differentiation undergoes "blockage" or "arrest" – or just stops – at the precursor cell stage [34,35]. To the prevailing consensus, the retention of the expressed differentiated state in hemoblastosis appears mysterious and even illogical.

On the basis of this significant difference, we can try to understand certain "oddities" in the behavior of the under-differentiated leukemia cells under the influence of various agents, especially those that stimulate the proliferation of normal non-hematopoietic cells. One "oddity" is that leukemia cells to a certain extent preserve the sensitivity towards regulatory mechanisms that are characteristic of normal cells. This allows the possibility of inducing differentiation in leukemia cells: a cell in which differentiation has been stopped can subsequently resume the process, up to the terminal stage, under the influence of various agents [35-37].

Most surprising, according to numerous data in the literature [38-40], is the ability of leukemia cells to differentiate under the influence of phorbol ethers. These compounds serve as growth stimulators and tumor promoters for the majority of normal non-hematopoietic cells. Through certain isoforms of protein kinase C (PKC), they launch the cycle of phosphatidylinositide, lipo- and cyclooxygenase signal pathways, which activate proliferation, with ROS increasing the formation of oxidized metabolites of arachidonic acid. This leads to an increased Δ(PO – AO) imbalance, generating a condition required for cell proliferation but not differentiation. Why then is an inverse picture observed in the leukemia cell lines HL-60, К-562, U-937 and others when they are exposed to the above-mentioned promoters, i.e. when the differentiating phenotype is induced? There is still no exact answer to this question; our views might be of some interest in this apparently contradictory situation.

Let us start from the following statement. The transfer to a "carcinogenous" Δ_C_(PO – AO) imbalance in non-hematopoietic cells is most likely to start during the proliferation stage, i.e. from the state that is commonly considered a prerequisite for tumor transformation. In hematopoietic cells, increase of the Δ_C_(PO – AO) imbalance to the Δ_L_(PO – AO) leukemogenic level frequently (but not always) begins directly during one of the early stages of differentiation. We consider this moment to be of primary importance. Differentiation is a multistage process, alternating with cell division. Many changes occur at each stage in transcription and translation, implementing a particular part of the differentiation program and creating the preconditions required for progression to the succeeding stage.

Mitochondria are again variously involved in the proliferation and differentiation of hematopoietic cells. They are quite dynamic structures, subject to regular fusion and fission during both biogenesis and its antithesis, disintegration, and at different stages of cell development. These processes inevitably change with the total capacity and activity of the mitochondria. In PSHC, which have few mitochondria, the processes in question probably occur on a limited scale. A well-known regularity is observed: an increased concentration of mitochondrial material is a prerequisite for the shift of balance from proliferation to differentiation [33,41].

During mitochondriogenesis, because O_2 _consumption grows, intracellular levels of pO_2 _and of the ROS and RNS signal molecules fall markedly and the Δ_P_(PO – AO) imbalance decreases to the range Δ_D_(PO – AO) required for differentiation of "specialized" functions. When these parameters change in such a way, transcription factors that depend on them are activated and the genes required for PSHC differentiation are expressed. Another opinion on this point is expressed by the authors of [5]. They suppose that NAD(P)H-oxidase is involved in hematopoietic stem cell differentiation. Fulfilling the role of free O_2 _sensor, this enzyme produces ROS, which as signal molecules induce the expression of genes needed for mitochondriogenesis, to which differentiation of these cells is linked. From the point of view of our "oxygen-peroxide" approach, this differentiation is again determined by reduction of the Δ_P_(PO – AO) imbalance at mitochondriogenesis in proliferating PSHC to the differentiating level Δ_D_(PO – AO). Subsequently, the same authors [42] demonstrated another phenomenon. Some catalytic subunits of the NADPH oxidase family, and all isoforms of its regulatory subunits, are expressed at the mRNA and protein levels in hematopoietic stem cells. These results may be interpreted in terms of fine adjustments of the ROS level, produced constituently, via positive feedback. This makes redox-mediated regulation of growth and differentiation possible in these stem cells.

All the events during mitochondrial degradation and destabilization develop in the opposite direction. Because O_2 _utilization falls in such mitochondria, pO_2 _rises, ROS and RNS concentrations are increased, and the Δ_D_(PO – AO) imbalance increases again to Δ_P_(PO – AO). Normally, these processes alternate until the hematopoietic cells reach terminal differentiation. Unfortunately, details of the triggering mechanism that regulates the interrelationship between proliferation and differentiation are still not clear. The events occurring here can develop, for instance, under the following scheme. At some *intermediate *stage of hematopoietic cell differentiation, when the Δ_D_(PO – AO) imbalance changes to the Δ_P_(PO – AO) range, active spontaneous change or the effects induced by pro-leukemogenic agents and factors starts. These further reduce the capacity and/or activity of the mitochondrial base. One may be involved in suppressing the set of nuclear genes that encode components of the mitochondrial protein complexes. Therefore, the Δ_D_(PO – AO) imbalance can immediately – bypassing the Δ_A1_(PO – AO) range – increase to the leukemogenic level Δ_L_(PO – AO), and the hematopoietic cell state is transformed towards uncontrolled proliferation. As at the Δ_P_(PO – AO) imbalance, this blocks differentiation at the stage of the *under-differentiated *precursor cell according to the non-antagonistic trigger principle. Such an effect is possible given the availability of genes "tuned" by a reversible trigger to fix the cell in one of two possible states. In the first state, leukemogenic proliferation at Δ_L_(PO – AO) interrupts differentiation. In the second, in contrast, differentiation at the Δ_D_(PO – AO) imbalance interrupts proliferation. This switch is performed under the influence of agents and factors that initially change the levels of prooxidants and antioxidants. The intervals between the "specialized" ranges of imbalances in hematopoietic cells are apparently insignificant. Therefore, transfers from one range to another occur comparatively easily, even with relatively small changes in the PO- and AO- constituents of the Δ(PO – AO) imbalance.

The gene HOX11 has interested us in this respect. According to the data described in [43], HOX11 is regarded as the gene causing leucosis. It is able to immortalize hematopoietic cell lines and is clinically associated with acute T-cell lymphoblastic leukemia in children. It has also been shown that enhanced HOX11 expression changes the erythroid differentiation of J2E cells towards the formation of less mature precursor cells. On the basis of these facts, the authors of this investigation [43] drew the following conclusion: disturbance of hematopoietic cell differentiation is responsible for the pre-leucosis immortalization of cells by HOX11 oncoprotein. These data could in our view be interpreted in another way. Specifically, we suspect that the HOX11 oncogene somehow – directly or indirectly – produces a prooxidant action. Therefore, the PO/AO imbalance in a cell with such an active oncogene increases to the leukemogenic level Δ_L_(PO – AO). The signal molecules corresponding to this proliferative level, including ROS, RNS and some peroxides, then induce the trigger mechanism of switching. This interrupts the process of differentiation that occurred before the cell was transferred into a leukemogenic state by the Δ_D_(PO – AO) imbalance. In the situation described, the effect on leukemia cells of factors that in some way lower the Δ_L _imbalance can lead to A1 apoptosis. A publication devoted to the induction of apoptosis in P388 lympholeucosis cells could serve as an example here [44]. A more marked reduction of the Δ_L _imbalance to the Δ_D _value should remove the differentiation blockade and differentiation should continue. In contrast, the effect on leukemia cells of agents and factors that enhance intracellular oxidative processes leads, in our view, to A2 apoptosis; for example, of HeLa and U-937 cells (see above).

Returning to the differentiating potential of phorbol ethers, we suggest the following. According to old information [45,46], these ethers, by activating PKC, enhance the phosphorylation of adenylate cyclase system components. This action increases the sensitivity and activity of adenylate cyclase, which is known to be low in leukemia cells. Further events within a leukemia cell may, according to our proposal, develop in the following sequence: increase in the cyclic adenosine monophosphate (cAMP) content → stimulation of cAMP-activity of mitochondria respiratory enzymes [47] → increased O_2 _consumption by these (relatively few) organelles → reduction of intracellular O_2 _and of the Δ_L_(PO – AO) imbalance to the differentiating level Δ_D_(PO – AO). The correspondingly reduced levels of the signal molecules, ROS and RNS, induce the activity of certain transcription factors and the expression of genes that depend on them, including genes required for differentiation.

The transfer proliferation → differentiation should obviously be accompanied by the suppression of most of the cell cycle control genes, and on the other hand by the expression of genes that inhibit this cycle. In particular, specific cyclin-dependent kinase inhibitors are expressed in the G_1 _phase, and these reprogram leukemia cells towards terminal differentiation [48]. The same regulatory mechanism may contribute to the production of endogenous bioregulatory myelopeptides, which can induce the terminal differentiation of HL-60 and К-562 cells and also stimulate *in vitro *differentiation of bone marrow cells in AML patients [49]. A sufficient mass of new information has been collected on the participation of different ROS and RNS as signal molecules in the simultaneous positive and negative regulation of expression of hundreds of genes, including oncogenes [50,51].

The foregoing reasoning about the differentiation of leukemia cells also applies to some non-hematopoietic tumor cells. They are also known to be differentiated, though in fewer cases.

**Figure 2:**
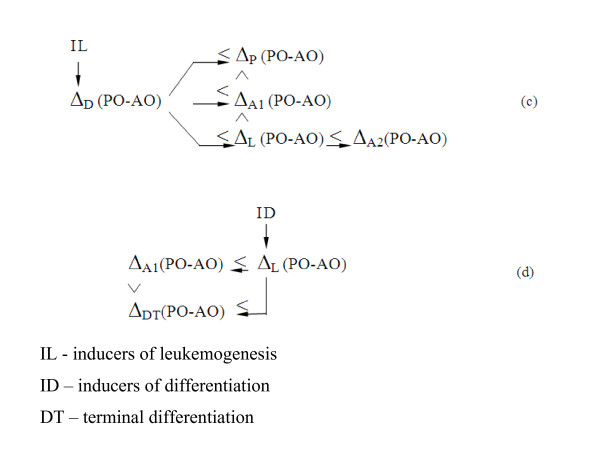
Expected transfers of "specialized" imbalances in the hematopoietic cells under the influence of inducers of leukemogenesis and differentiation

Overall, taking into account the normal exchange between proliferative and differentiating imbalances, Δ_P _↔ Δ_D_, the "specialized" imbalances Δ(PO – AO) applied to the transformation and differentiation of hematopoietic cells are presumably linked by inequalities (see figure 2)

In the form presented here, the oxygen-peroxide concept of leukemogenesis cannot reasonably explain the facts described in the literature on the differentiation of leukemia cells under prooxygenase effects. For example, differentiation of leukemia cells is induced by dimethyl sulfoxide, tetradecanoyl phorbol-acetate (TPA) and dibutyryl-cAMP by increasing ROS formation. These data do not quite fit the scheme we present.

## Conclusion

We have related our discussion of the oxygen-peroxide mechanism of leukemogenesis to the fundamental fact that there are only a few mitochondria in PSHC, the precursors of the various blood cell lines. The very existence of mitochondria, the main consumers of O_2 _entering the cells, allows us to regard these ATP-producing organelles as the major anti-oxygen stage in the cell's protective antioxidative system. Variation of the mitochondrial base capacity (quantity and quality of mitochondria) is considered an important and particularly effective channel for regulating the oxidative stress level within a cell. The ideas used in the present article are centered on the adaptive emergence of a sequence of "specialized" ranges of Δ(PO – AO) imbalance during the course of evolution and the fixation of these ranges in cells. Each entails the possibility and even necessity of a implementing a definite complex biochemical process.

The principal important effects arise from the minimization of the mitochondrial content of hematopoietic stem cells. Taken together, they result in hematopoietic stem cells becoming objects at high risk of spontaneous or induced transformation into leukemia cells via the oxygen-peroxide mechanism (figure 1). The anti-leucosis actions of various antioxidants (resveratrol etc.) support the view that leucosis proceeds in accordance with this mechanism. The reasoning presented in this article leads to inferences about the causes of congenital and children's leucosis, and several facts about leucosis being induced by definite agents (vitamin K_3_, benzene, etc.) are interpreted in a new way.

The capacity of leukemia cell differentiation to be blocked at the precursor cell stage still remains mysterious. According to our version, the transformation of normal non-hematopoietic cells into tumor cells begins during the proliferation stage. But normal hematopoietic cells are transferred to the leucosis state by the effects of leukemogenic agents and factors starting from one of the intermediate stages in differentiation, and they stop at that stage. In the presence of appropriate stimuli, the under-differentiated leukemia cell can continue to differentiate to its terminal stage.

Normally, the switch from proliferation to differentiation and back occurs under the trigger principle. The Δ(PO – AO) imbalances corresponding to these two processes are involved in this switching; for normal cells, Δ_D _< Δ_P_, and for leucosis cells, Δ_D _< Δ_L_. The agents (antioxidants, etc.) that lower the Δ_L _imbalance in a leukemia cell to the Δ_A1 _value or directly to Δ_D _induce type A1 apoptosis or differentiation respectively. An increase in the Δ_L _imbalance to the Δ_A2 _level leads to A2 type apoptosis in a leukemia cell (see inequalities c and d). In all cases, the direct and/or indirect participants in the switches are ROS, RNS, some peroxides and "specialized" Δ(PO – AO) imbalances corresponding to their levels.

To validate the hypothesis discussed here, specific research directions are required. Success depends on the development of precise and accessible methods for measuring and assessing the oxidative stress level in various cell types in general, and hematopoietic cells in particular, in their different functional states.

## Competing interests

The authors declare that they have no competing interests.

## Authors' contributions

BL and SBI made the main contributions to this paper. All authors read and approved the final manuscript.
